# Designing and Introducing a New Artificial Feeding Apparatus for Sand Fly Rearing

**Published:** 2018-12-25

**Authors:** Mahboubeh Fatemi, Zahra Saeidi, Parviz Noruzian, Amir Ahmad Akhavan

**Affiliations:** 1 Department of Medical Entomology and Vector Control, School of Public Health, Tehran University of Medical Sciences, Tehran, Iran; 2 Tajhiz Gostar Omid Iranian Company, Tehran, Iran

**Keywords:** *Phlebotomus papatasi*, Artificial feeding apparatus, Blood-feeding insects, Sand fly

## Abstract

**Background:**

Due to strict ethical rules, the risk of accidental disease transmission and the most importantly, inconvenience regarding using of live animals, artificial feeding apparatus has been developed for colonization of haematophagous insects. Rearing of sandfly is more difficult than other haematophagous insects.

**Methods:**

In the current study, a new apparatus for membrane feeding of *Phlebotomus papatasi* was designed, made and compared with available apparatus in Sand Fly Insectary, Tehran University of Medical Sciences, Tehran, Iran, in 2014.

**Results:**

In comparison to other apparatus designed for artificial feeding of other arthropods, our designed apparatus had the highest performance which after up to 1h, the majority of sand flies landed and took blood and among tested membranes, chicken skin was proved the most efficient membrane.

**Conclusion:**

Sand fly artificial feeding apparatus can be used at least for rearing of *Ph. papatasi*.

## Introduction

Insect colonization has been intensified by increasing of interest in understanding of their life cycle, physiology, anatomy, genetics, and study on arthropod-borne diseases, insecticide and repellency tests to control the diseases (1). Blood-feeding is one of the most critical and difficult steps in rearing process of haematophagous insects. Animal maintenance for blood feeding of the colony, not only is costly and time-consuming but also requires ethical considerations. Due to these restrictions, risk of accidental disease transmission and inconvenience of using live animals, artificial feeding apparatus has been developed. Artificial feeding is a useful technique in nutritional and behavioral studies on insects such as feeding stimulants and interaction between parasite and invertebrate host (2). Moreover, one of the most advantages of this technique in the parasite and vector interaction studies is capability of adding a certain concentration of parasite to feeding mixture (3). Precise control over the test conditions is considered as another advantage of this method compared to feeding on live hosts. Based on advantages mentioned above, membrane feeding is preferred to feeding on animals.

The earliest attempt at feeding tsetse flies through rat skin was made by Rodhain et al. (4). Subsequently, this technique has been used successfully to feed various haematophagous insects including vectors and their transmittable pathogens to study developmental stages and transmission cycle of micro-organism inside their body (5–15). Establishment and maintenance of sandflies are far more difficult than other haematophagous insects (16–17).

The aim of the current study was to design and introduce a new apparatus for artificial feeding of *Phlebotomus papatasi* in the insectary condition.

## Materials and Methods

In order to membrane feeding of *Ph. papatasi* four artificial feeding apparatus including: Tick artificial feeding apparatus, Mosquito artificial feeding apparatus based on the idea of Cosgrove et al. (18), modified Cosgrove et al. apparatus and a new artificial feeding apparatus which was designed and made in this study in Sand Fly Insectary, Tehran University of Medical Sciences, Tehran, Iran, in 2014, (The patent number: 86836, Islamic Republic of Iran) were compared.

**Fig. 1.**
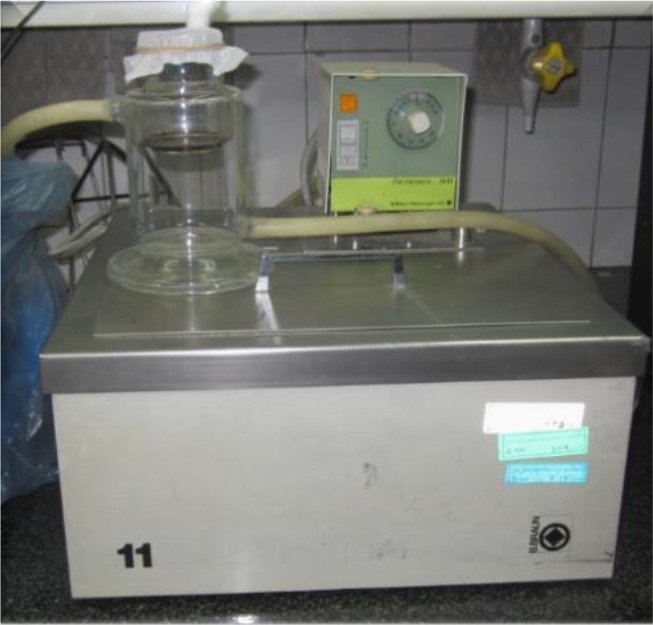
Tick artificial feeding apparatus

Tick artificial feeding apparatus has a chamber to hold the blood, 5.5cm in diameter and 12.5cm deep. The minimum required volume of blood is 5ml. It was connected to a water bath that circulates warm water inside the apparatus. A glass tube was oriented vertically in the chamber that is 4.5×7cm. Membranes were stretched over one end of the glass tube which is in contact with blood mixture. Fine mesh gauze is fitted on another end of the apparatus to transfer sand flies through it (Fig. 1).

**Fig. 2.**
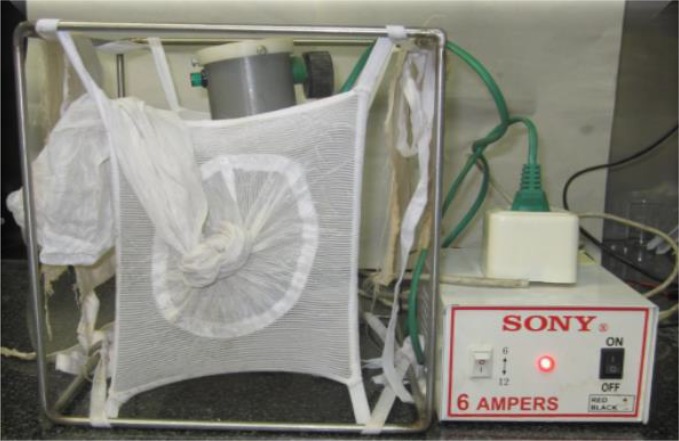
Mosquito artificial feeding apparatus of based on the idea of Cosgrove et al.

The second apparatus consisted of an 8×7 cm aluminum chamber with a volume of 2.8 ml heated by electrical elements. A thermostat has been connected to adjust the desired temperature (Fig. 2).

**Fig. 3.**
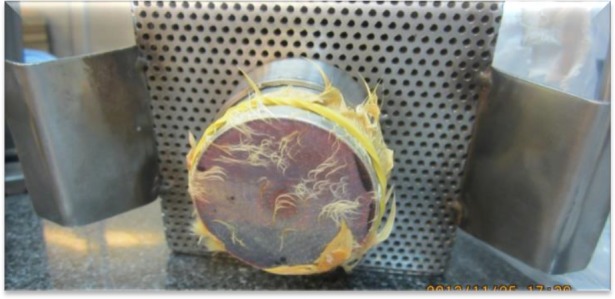
Modified Cosgrove’s apparatus

Chamber in modified Cosgrove et al. apparatus has a volume of 9ml and the temperature is displayed on a digital screen that is adjustable with buttons (Fig. 3).

The idea of the design of sand flies artificial feeding apparatus was inspired by the two previously available equipment (19, 20). The apparatus contains a stainless steel container in a volume of 6L and a steel plate embedded on the top of the container. The steel plate is used as a container for a water bath if needed. To assure a constant temperature in the blood feeders, a pair of water outlet and inlet pipes was designed for each blood feeder of the apparatus.

**Fig. 4.**
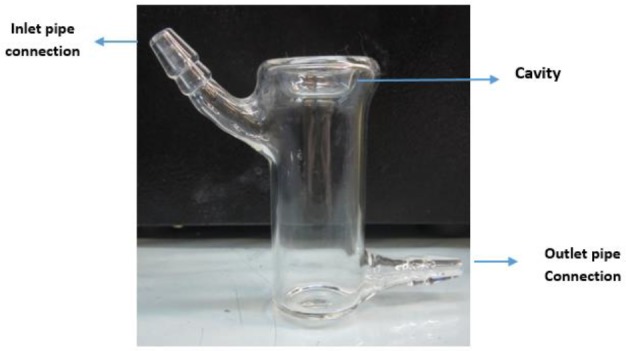
Glass blood feeder

In order to offer blood supply for the insects, 7.5×2.5cm glass blood-feeders were designed (Fig. 4). A cavity at the top was designed to hold the blood mixture (up to 2ml) and a pair of water outlet and inlet pipes for circulating warm water inside the blood-feeder. In each experiment, up to four blood-feeders can be connected to apparatus at the same or different angles, simultaneously. All the pipes were considered 120cm in length in order to make the same condition.

**Fig. 5.**
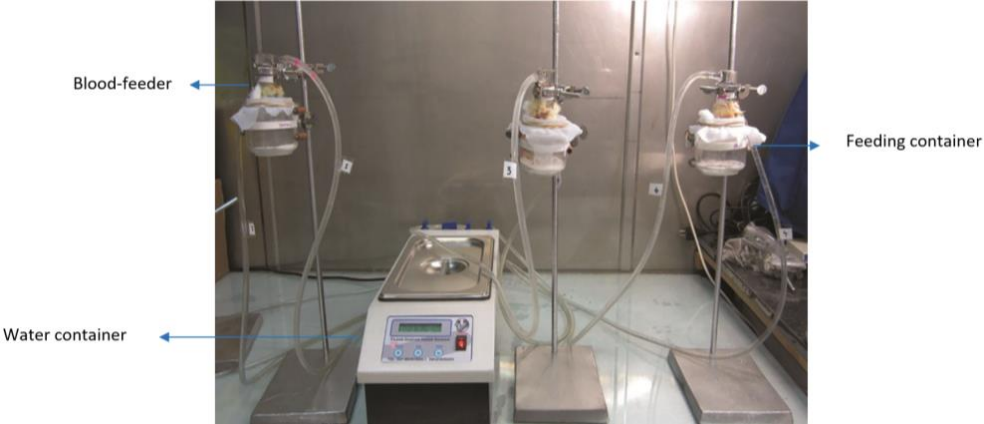
*Phlebotomus papatasi* artificial feeding apparatus

A temperature sensor was placed inside the outlet, and a built-in display installed to monitor experiment process. The temperature and time were adjustable with buttons on the screen. The Alarm sound could be heard after finishing the time given to apparatus (Fig. 5). To mimic natural condition of blood feeding, the membrane temperature was adjusted to 35±1 °C.

The bottom of feeding containers (5.5cm height, 4cm diameter) was moistened with distilled water before the introduction of the sand flies and placed in close contact with the membrane by the clamp. Therefore, the membrane is accessible to the sand flies for blood-feeding.

In four mentioned artificial feeding apparatus, Parafilm, Nescofilm and skin of three-week-old chicken as membrane were tested and compared. Membranes were stretched over the blood mixtures in one end of each feeder and fastened with elastic band.

In each case, a new membrane and fresh blood were used and *Ph. papatasi* were starved for 2h before the blood meal. Moreover, three sources of defibrinated or heparinized blood of human, BALB/c mice and *Rhombomys opimus* were used to achieve the highest blood feeding. All experiments were carried out under the same condition at 26±2 °C temperature and 80 % relative humidity.

Animal protocol was approved by the Ethics Committee of Tehran University of Medical Sciences, Tehran, Iran.

## Results

In tick and Cosgrove’s artificial feeding apparatus, no sand fly landed to blood chamber.

In modified Cosgrove’s artificial feeding apparatus, the sand flies landed on the membranes but no *Ph. papatasi* took blood. In three above-mentioned apparatuses, because of presence of only one blood feeder, each blood sample was tested separately. In sand fly artificial feeding apparatus made in the current study, after up to 1h the majority of sand flies landed and took blood. Moreover, three blood sources were checked simultaneously.

Among the tested membranes, the chicken skin was the best and the heparinized blood had the most efficient because it was not clotted even after 4h. No preference among the tested blood was observed.

## Discussion

Feeding through membrane has been utilized for various hematophagous arthropods to purpose of mass rearing of insects or to artificially infect vectors with parasites (3, 5, 18, 20–22). There were disadvantages in the available artificial feeding equipment such as the temperature which could only be measured in the water tank with no temperature sensor in the vicinity of membrane, and also the time that was not adjustable. The current new designed feeding apparatus does not have the above-mentioned weakness points. Perhaps the lack of circulation of warm water and the moisture, that is critical for sand fly feeding, were responsible for unattractiveness of blood chamber for *Ph. papatasi* in modified and Cosgrove’s artificial feeding apparatus.

Each blood-sucking arthropod has a preference host to take blood, determining the most appropriate blood lead to the highest blood feeding that has direct effect on egg production. Because of capability of supporting several blood feeder by this apparatus various blood sources can be tested simultaneously to achieve the best result. Results of artificial-feeding of *Ph. papatasi* showed no significant differences in blood-feeding proportion of sand flies between human, BALB/c mice, and *Rh. opimus.* In agreement with our results, there was no preference between human blood and blood from different types of host (cow, dog, guineapig, hamster, horse, pig, rabbit) in *Ph. papatasi* blood feeding (23).

Angle of blood-feeder can affect on percent of *Lutzomyia shannoni* blood feeding (20). Therefore, ability to change the angle on this device has been provided.

Besides, different kinds of membrane including natural or synthetic such as guinea pig mesentery and large bowel, batwing, human peritoneum, human kidney capsule, Ox liver capsule, Baudruche, chick skin, Parafilm, Chitosan, dialyzing bag and so on have been tested to stimulate insects to taking a blood-meal through membrane (8, 19, 24–26). However, different studies, same to our study, have shown that chicken skin is more effective than other membranes (25, 27, 22, 28).

## Conclusion

New designed artificial feeding apparatus could be used for rearing of *Ph. papatasi* instead of live animals.
